# An Epitome of Braithwaite’s Retrospect

**Published:** 1860-11

**Authors:** 


ART. XII.—*An Epitome of Braithwaite's Retrospect of Practical Medicine and Surgery.* By WALTER S. WELLS, M.D. New York, 1860.

THE sixth and last part of this work has just been placed upon our table. We congratulate Dr. Wells upon the completion of his self-imposed task, and take great pleasure in recommending his Epitome to the favorable notice of the profession. Comprising, as it does, a faithful abstract of one of the most important periodicals of the day, it cannot fail to prove of immense service to the practitioner, who, in the busy routine of his arduous labors, has but little time to read the original work, now numbering many volumes.

We are happy to see that Dr. Wells proposes to continue the Epitome, in conjunction with the best journals of the age, under the title of "Summary of Medical Science," embodying, as the name implies, the pith of the more important articles in the various medical essays emanating from the leading medical men throughout the world.

